# UDP-Glucosyltransferases from Rice, *Brachypodium*, and Barley: Substrate Specificities and Synthesis of Type A and B Trichothecene-3-*O*-β-d-glucosides

**DOI:** 10.3390/toxins10030111

**Published:** 2018-03-06

**Authors:** Herbert Michlmayr, Elisabeth Varga, Alexandra Malachová, Philipp Fruhmann, Marta Piątkowska, Christian Hametner, Jana Šofrová, Günther Jaunecker, Georg Häubl, Marc Lemmens, Franz Berthiller, Gerhard Adam

**Affiliations:** 1 Department of Applied Genetics and Cell Biology, University of Natural Resources and Life Sciences, Vienna, (BOKU), Konrad Lorenz Str. 24, 3430 Tulln, Austria; gerhard.adam@boku.ac.at; 2 Department of Food Chemistry and Toxicology, University of Vienna, Währinger Str. 38, 1090 Vienna, Austria; 3 Christian Doppler Laboratory for Mycotoxin Metabolism and Center for Analytical Chemistry, Department of Agrobiotechnology (IFA-Tulln), BOKU, Konrad Lorenz Str. 20, 3430 Tulln, Austria; elisabeth.varga@boku.ac.at (E.V.); alexandra.malachova@boku.ac.at (A.M.); marta.piatkowska@boku.ac.at (M.P.); sofrova@email.cz (J.Š.); franz.berthiller@boku.ac.at (F.B.); 4 Institute of Applied Synthetic Chemistry, Vienna University of Technology, Getreidemarkt 9/163, 1060 Vienna, Austria; philipp.fruhmann@tuwien.ac.at (P.F.); christian.hametner@tuwien.ac.at (C.H.); 5 CEST Kompetenzzentrum für elektrochemische Oberflächentechnologie GmbH, Viktor-Kaplan-Str. 2, 2700 Wiener Neustadt, Austria; 6 Department of Chemistry and Biochemistry, Mendel University in Brno, Zemědělská 1, 613 00 Brno, Czech Republic; 7 Romerlabs Division Holding GmbH, Technopark 1, 3430 Tulln, Austria; jaunecker@romerlabs.com (G.J.); georg.haeubl@romerlabs.com (G.H.); 8 Biotechnology in Plant Production, IFA-Tulln, BOKU, Konrad Lorenz Str. 20, 3430 Tulln, Austria; marc.lemmens@boku.ac.at

**Keywords:** Fusarium head blight, phase II detoxification, masked mycotoxin, NMR, cereals

## Abstract

Trichothecene toxins are confirmed or suspected virulence factors of various plant-pathogenic *Fusarium* species. Plants can detoxify these to a variable extent by glucosylation, a reaction catalyzed by UDP-glucosyltransferases (UGTs). Due to the unavailability of analytical standards for many trichothecene-glucoconjugates, information on such compounds is limited. Here, the previously identified deoxynivalenol-conjugating UGTs HvUGT13248 (barley), OsUGT79 (rice) and Bradi5g03300 (*Brachypodium*), were expressed in *E. coli*, affinity purified, and characterized towards their abilities to glucosylate the most relevant type A and B trichothecenes. HvUGT13248, which prefers nivalenol over deoxynivalenol, is also able to conjugate C-4 acetylated trichothecenes (e.g., T-2 toxin) to some degree while OsUGT79 and Bradi5g03300 are completely inactive with C-4 acetylated derivatives. The type A trichothecenes HT-2 toxin and T-2 triol are the kinetically preferred substrates in the case of HvUGT13248 and Bradi5g03300. We glucosylated several trichothecenes with OsUGT79 (HT-2 toxin, T-2 triol) and HvUGT13248 (T-2 toxin, neosolaniol, 4,15-diacetoxyscirpenol, fusarenon X) in the preparative scale. NMR analysis of the purified glucosides showed that exclusively β-d-glucosides were formed regio-selectively at position C-3-OH of the trichothecenes. These synthesized standards can be used to investigate the occurrence and toxicological properties of these modified mycotoxins.

## 1. Introduction

**Figure 1:**
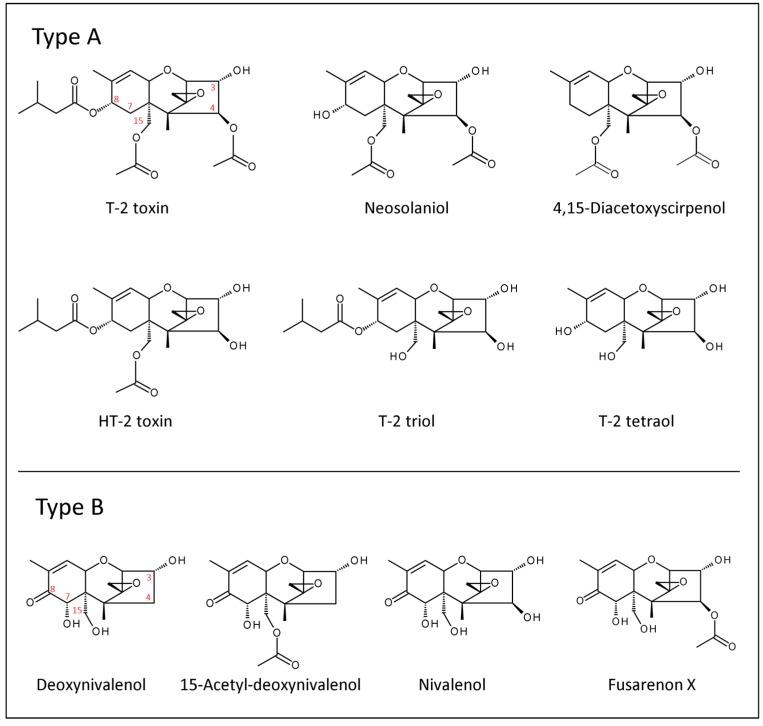
Chemical structures of important trichothecenes.

Trichothecenes are a large group of toxic fungal secondary metabolites with a common tricyclic 12,13-epoxytrichothec-9-ene core structure. They are produced by several fungal genera of the order *Hypocreales*, and their primary mode of action is the inhibition of eukaryotic protein synthesis [1]. Trichothecenes are confirmed (deoxynivalenol, DON) or suspected virulence factors of *Fusarium* species that cause severe diseases of crop plants such as Fusarium head blight (FHB) of small grain cereals [1,2,3]. Species belonging to the *Fusarium graminearum* complex mainly produce type B trichothecenes such as deoxynivalenol, the most frequent trichothecene toxin worldwide, and nivalenol (NIV), which is most prevalent in Asia and South America [4]. The often co-occurring acetylated derivatives 3-acetyl-DON, 15-acetyl-DON, and 4-acetyl-NIV (usually referred to as fusarenon X, FUSX) are biosynthetic precursors of DON and NIV [1]. *F. sporotrichioides*, *F. langsethiae*, and *F. poae* are common producers of the type A trichothecenes T-2 toxin (T2) and HT-2 toxin (HT2). T2 and HT2 are among the most toxic trichothecenes [5] and often co-occur because HT2 (“hydrolyzed T2”) is also a degradation product of T2 lacking the C-4 acetyl-group [6]. Further type A trichothecenes produced by these species are neosolaniol (NEO) and 4,15-diacetoxyscirpenol (DAS) [7]. The structures of the most relevant trichothecene toxins are shown in Figure 1.

Cereal crop plants exhibit varying degrees of susceptibility/resistance to *Fusarium* infections. Resistance to initial infection is referred to as type I resistance, resistance by inhibited or reduced disease spread as type II resistance. In wheat, several quantitative trait loci (QTL) conferring partial resistance are known [8], one of the most effective being *Fhb1* on chromosome 3B (type II resistance) [9]. Trichothecenes are regarded as the main *Fusarium* virulence factors promoting disease spread, and therefore it is plausible to assume that the ability to detoxify or eliminate trichothecenes should provide a certain level of (i.e., type II) resistance. 

Xenobiotic metabolism in plants is divided into three general phases [10,11]. Phase I involves a number of reactions that increase polarity and expose or introduce functional groups such as hydroxyl-groups. Typical phase I modifications of trichothecenes involve the hydrolysis of acetylated derivatives, e.g., of T2 to HT2. While phase I metabolites are not necessarily less toxic than their precursors, conjugation with hydrophilic molecules in phase II usually results in less toxic metabolites. The major pathways are glycosylation catalyzed by UDP-glycosyltransferases (UGTs) and glutathione conjugation catalyzed by glutathione-S-transferases. DON-3-*O*-β-d-glucoside (DON-3-Glc) is so far the best documented phase II metabolite of DON, but sulfate-, glutathione-, and cysteine-conjugates were also reported [12,13,14]. In phase III (sequestration), metabolites are transported to the vacuole or to the apoplast. 

To which extent such detoxification systems contribute to effective resistance is a matter of controversy, as resistance is complex, and it is far from trivial to pinpoint a specific mechanism. This is also evident in the case of the resistance QTL *Fhb1*, which has first been associated with the ability to detoxify DON to DON-3-Glc [15]. However, that DON-detoxification is directly responsible for resistance has been refuted, and mechanisms like cell wall thickening due to deposition of hydroxycinnamic acid amides, phenolic glucosides, flavonoids [16], and, recently, a pore forming toxin-like protein [17] were identified. Nevertheless, analysis of near isogenic wheat lines confirmed that lines harboring *Fhb1* show significantly elevated DON-3-Glc/DON ratios [18]. While fine mapping did not reveal any candidate DON-conjugating UGTs encoded in the *Fhb1* locus [19], the same study clearly demonstrated that *Fhb1* does reduce sensitivity to DON (see also Supplemental Figure S5 in reference [19]). Since the pore-forming toxin gene has no effect on DON-detoxification [17], more than one gene in the *Fhb1* interval seems to contribute to *Fusarium* resistance.

An undisputable consequence of plant detoxification systems is that cereal products often contain toxin derivatives that have previously been, or perhaps still are, unknown. The term “masked mycotoxins” has been coined to express that such toxins are not captured by routine analyses, although it cannot be excluded that they are toxicologically relevant [20,21]. Meanwhile, it has been recommended to use this term only for metabolites that originate from the plant metabolism, while toxin derivatives of fungal origin (e.g., biosynthetic precursors) fall into the more general category “modified toxins” [22]. The main open questions related to this issue are those addressing occurrence and the toxicological relevance of masked/modified mycotoxins. While they are generally assumed to be less toxic than the parent compounds *per se*, it is unclear to which extent they add to the toxic load of food/feed. Due to the lack of toxicity data, the CONTAM panel of the European Food Safety Authority (EFSA) has recommended assuming the same toxicity for modified forms as for their parent toxins [23]. Most information so far is available on the phase II metabolite DON-3-Glc, which has been detected in wheat, oats, barley, and corn samples worldwide with high variations in incidence and concentration [24]. A recent review reported an incidence of 55% and an average of 85 µg/kg DON-3-Glc in unprocessed cereal samples [25]. DON-3-Glc does not inhibit protein synthesis [26] and is probably of low acute toxicity. However, it is almost completely hydrolyzed to DON in the intestinal tracts of rats and pigs and may therefore be relevant after all, although hydrolysis occurs late in the intestinal tract [25,27,28]. 

Glucosylated forms of NIV [29,30,31], FUSX [29], T2, and HT2 [32,33] have also been identified in cereal samples. Due to the lack of commercial analytical standards, occurrence data are very limited in these cases. It is therefore important to obtain such compounds in pure form for analytical use and, if relevant, to assess their toxicity, e.g., in feeding trials. We therefore aimed to synthesize the β-glucosides of a range of relevant trichothecenes using previously identified plant UGTs. A series of transcriptomic analyses of barley [34,35,36] identified a DON inducible UGT (HvUGT13248) converting DON to DON-3-Glc [37]. It was later shown that over-expression of the HvUGT13248 gene in *Arabidopsis thaliana* and wheat leads to reduced sensitivity to both DON and NIV, and limits FHB disease spread [38,39,40]. A further study [41] investigated homologs of HvUGT13248 and identified enzymes from rice (OsUGT79) and *Brachypodium distachyon* (Bradi5g03300) that are also capable to glucosylate DON. All of these possess high sequence similarities, belong to clade L/family 74 in the *Arabidopsis* nomenclature of UGTs [42], and are evolutionary related [41]. A common characteristic of these enzymes is regioselective glucosylation of DON at C-3-OH. Recently, compelling evidence has been presented that Bradi5g03300 is involved in *Fusarium* resistance of *B. distachyon.* Mutations in the gene increased root sensitivity to DON and susceptibility to *F. graminearum*, while over-expression conferred increased root tolerance to DON and spike resistance to the fungus [43]. Due to its relatively good expression in *Escherichia coli*, OsUGT79 was previously used for the preparative syntheses of DON-3-Glc and NIV-3-Glc [38,44]. In the present paper we extend previous work by demonstrating the glucosylation of several trichothecene toxins (15-Ac-DON, T2, HT2, T2 triol, DAS, NEO, FUSX) using OsUGT79 and HvUGT13248. Furthermore, a biochemical analysis was conducted to better understand the substrate specificities of these UGTs. 

## 2. Results and Discussion

### 2.1. Substrate Specificities of Trichothecene-Conjugating UGTs

Recombinant OsUGT79 was previously identified as an efficient catalyst for DON-3-Glc and NIV-3-Glc synthesis. As shown below, it is also active with HT2 and T-2 triol (T2 triol), and could be applied to synthesize HT2-glucoside and T2 triol-glucoside. Unfortunately, it was impossible to glucosylate C-4 acetylated toxins (T2, FUSX, DAS, and NEO) with OsUGT79. Although the masses of such glucosides were detectable by liquid chromatography coupled to high resolution mass spectrometry (LC-HRMS), even at high enzyme concentrations and long incubation times, the acceptor concentrations remained unchanged and preparative synthesis was out of reach. This is in agreement with two recent papers on structure, catalytic mechanism, and substrate binding of OsUGT79 [45,46] that reported OsUGT79 is inactive with T2 and FUSX. 

We further investigated HvUGT13248, which prefers NIV over DON [38], implying that the enzyme may possibly be more prone to accept substrates with C-4 substitutions. Unfortunately, HvUGT13248 is recalcitrant to heterologous expression in *Escherichia coli*. Protein expressed with *E. coli* Rosetta (as done with OsUGT79) was inactive. We found that strain SHuffle^®^ T7 Express *lysY* (New England Biolabs, Frankfurt am Main, Germany), a BL21(DE3) derivative engineered to allow cytoplasmic disulfide bond formation, is capable of producing active HvUGT13248. This fact was surprising because such UGTs are usually cytosolic [47] and should not require disulfide bonds. A cytoplasmic disulfide bond isomerase expressed by this strain might be responsible for correct protein folding [48]. Furthermore, we have previously expressed this enzyme in fusion with a maltose binding protein (MalE) and *N*-terminal His_6_-tag, because this was a successful strategy with OsUGT79. Yet, the constructed *n*His_6_-MalE-HvUGT13248 was unstable and most of the active protein was lost during purification. Expression of a construct with *C-*terminal His-tag improved the yield after purification and contrary to OsUGT79, in which *N*-terminal fusion to the maltose binding protein improved specific activity approximately 10-fold, the best results with HvUGT13248 were obtained when the solubility tag was omitted. Still, the enzyme (HvUGT13248-*c*His_6_) was expressed at a very low level (estimated <5 mg per L broth), resulting in poor yield and impure protein after one-step purification. Nevertheless, this protein showed activity with C-4 acetylated trichothecenes and could be used to synthesize mg amounts thereof (see Section 2.2).

The specific activities of OsUGT79, HvUGT13248, and Bradi5g03300 were compared on a number of trichothecene toxins. The activities were determined by detecting the released UDP in a luciferase coupled assay (UDP-Glo; Promega, Fitchburg, WI, USA). The results (Table 1) imply generally low or absent capacities of glucosylate C-4 acetylated trichothecenes. OsUGT79 and Bradi5g03300 showed similar activity profiles and were inactive with T2, NEO, DAS, and FUSX. The low activity of OsUGT79 implied with FUSX could not be confirmed by LC-HRMS analysis. HvUGT13248 possesses the broadest substrate range and was able to conjugate all included toxins to varying degrees but also, here, the lowest activities were observed with the C-4 acetylated compounds. All enzymes were also active with zearalenone, another *Fusarium* toxin. The fact that only HvUGT13248 was also active with the flavonoids kaempferol and quercetin further confirms its rather unselective nature. 

HvUGT13248 and Bradi5g03300 showed their highest activities with the type A trichothecenes HT2 and T2 triol. OsUGT79 displayed about equal activities with DON, HT2, T2 triol, and T2 tetraol. An approximately two-fold catalytic efficiency with HT2 compared to DON was previously reported for OsUGT79 [46]. Here, Michaelis-Menten kinetics were determined with HvUGT13248 and Bradi5g03300 using DON, NIV, and HT2 as substrates (Table 2). As both enzymes were not pure after one-step affinity purification, *k*_cat_ was not calculated. The quotient of *V*_max_ over *K*_M_ was calculated in analogy to *k*_cat_/*K*_M_ to provide a relative analysis of catalytic efficiencies. The results obtained for HvUGT13248 with the UDP-Glo assay gave similar results for DON and NIV as previously determined with end-point assays followed by liquid chromatography coupled to tandem mass spectrometry (LC-MS/MS) measurements to quantify the formed glucosides [38]. This confirms an about 5-fold preference of NIV over DON and shows high efficiency with HT2 with about 30-fold catalytic efficiency estimated relative to DON (Table 2). With Bradi5g03300, we observed a *K*_M_ value of 0.37 mM with DON, which is far above the value of 32.5 µM previously reported [43]. Such differences may lie in use of different assays or different kinetic models used for data analysis. Our results imply that Bradi5g03300 prefers DON over NIV and shows its highest efficiency with HT2. 

### 2.2. Preparative Synthesis of Glucosides with OsUGT79 and HvUGT13248, and Purification

The different glucosides were synthesized over a long time period, and therefore the conditions of the individual batches vary considerably; detailed information on each batch is provided in Table 3. In general, the glycosylation reactions were performed at pH 7, with an excess of UDP-glucose and ≥1 mg/mL of one-step purified UGT (OsUGT79 or HvUGT13248). As mentioned above, batch syntheses of HT2-glucoside and T2 triol-glucoside with OsUGT79 were unproblematic. We were further able to glucosylate about 5 mg of each FUSX, T2, NEO, and DAS using HvUGT13248. Here, UDP-glucose was recycled with sucrose and sucrose synthase to improve reaction efficiency and achieve complete conversion [44]. In case of NEO, two peaks of formed glucosides were observed by LC-MS/MS. The first peak was later on identified as NEO-3-*O*-β-d-glucoside and the second one as the equivalent glucoside of 15-deacetyl-8-acetyl-neosolaniol (=iso-neosolaniol, iso-NEO). The aglycon whose isolation from *Fusarium tricinctum* culture was described before [49] was also identified in the NEO preparation used for the glucosylation. 

### 2.3. Characterization by HRMS, MS/HRMS, and NMR

After purification, the respective glucosides were characterized by LC-HRMS, and the proposed sum formulas were confirmed. The ammonium- or sodium-adducts were the most abundant ion species in positive ionization mode, and the formate-adducts were predominant in negative mode for all glucosides, whereas the quasi-molecular ions were hardly detectable: 15-Ac-DON-Glc (C_23_H_32_O_12_; [M + NH_4_]^+^ 518.2205, Δm = −4.2 ppm; [M + Na]^+^ 523.1769, Δm = −3.2 ppm; [M + HCOO]^−^ 545.1866, Δm = 1.8 ppm), HT2-Glc (C_28_H_42_O_13_; [M + NH_4_]^+^ 604.2960, Δm = 0.3 ppm; [M + Na]^+^ 609.2506, Δm = −1.9 ppm; [M + HCOO]^−^ 631.2581, Δm = 4.2 ppm), T2 tetraol-Glc (C_21_H_32_O_11_; [M + NH_4_]^+^ 478.2277, Δm = −0.1 ppm; [M + Na]^+^ 483.1817, Δm = −4.1 ppm; [M + HCOO]^−^ 505.1914, Δm = 2.5 ppm), T2 triol-Glc (C_26_H_40_O_12_; [M + NH_4_]^+^ 562.2847, Δm = −1.0 ppm; [M + Na]^+^ 567.2400, Δm = −2.1 ppm; [M + HCOO]^−^ 589.2481, Δm = 3.5 ppm), FUSX-Glc (C_23_H_32_O_13_; [M + NH_4_]^+^ 534.2173, Δm = −0.5 ppm; [M + Na]^+^ 539.1717, Δm = −3.4 ppm; [M + HCOO]^−^ 561.1814, Δm = 1.9 ppm), T2-Glc (C_30_H_44_O_14_; [M + NH_4_]^+^ 646.3074, Δm = 1.6 ppm; [M + Na]^+^ 651.2617, Δm = −1.0 ppm; [M + HCOO]^−^ 673.2700, Δm = 1.9 ppm), DAS-Glc (C_25_H_36_O_12_; [M + NH_4_]^+^ 546.2540, Δm = 0.1 ppm; [M + Na]^+^ 551.2095, Δm = −0.7 ppm; [M + HCOO]^−^ 573.2165, Δm = 4.2 ppm), NEO-Glc (C_25_H_36_O_13_; [M + NH_4_]^+^ 562.2497, Δm = 1.5 ppm; [M + Na]^+^ 567.2050, Δm = 0.3 ppm; [M + HCOO]^−^ 589.2118, Δm = 3.4 ppm), iso-NEO-Glc (C_25_H_36_O_13_; [M + NH_4_]^+^ 562.2482, Δm = −1.2 ppm; [M + Na]^+^ 567.2040, Δm = −1.4 ppm; [M + HCOO]^−^ 589.2126 Δm = 2.0 ppm). 

Further LC-MS/HRMS-measurements showed mainly the cleavage of the glucosidic bond and further fragmentation of the aglycon (loss of water, acetic, and isovaleric acid moieties, as well as the epoxy-group) (see Supplemental Figures S1–S3). Even at low collision energies the only visible fragments higher than the aglycon were the fragments equivalent to the protonated ions in case of HT2-Glc and T2 triol-Glc. Based on the LC-MS/HRMS data, it is not possible to predict where the glucose is attached to the trichothecene backbone. NEO-Glc and iso-NEO-Glc behave differently, and a quite distinct fragment with a mass of *m*/*z* 203.1065 is visible in the iso-NEO-Glc spectrum. It could be explained by the cleavage of the glucose moiety, the two acetyl-groups, the epoxide ring, and the C-15 CH_2_OH-group. Iso-NEO-Glc seems not to be as stable as NEO-Glc, which was also observed during NMR-analysis. There, the C-8 acetyl-group was partially cleaved off, and the structure was identified as a mixture of iso-NEO-3-*O*-β-d-glucoside and 8-deacetyl-iso-NEO-3-*O*-β-d-glucoside. 

**Figure 2:**
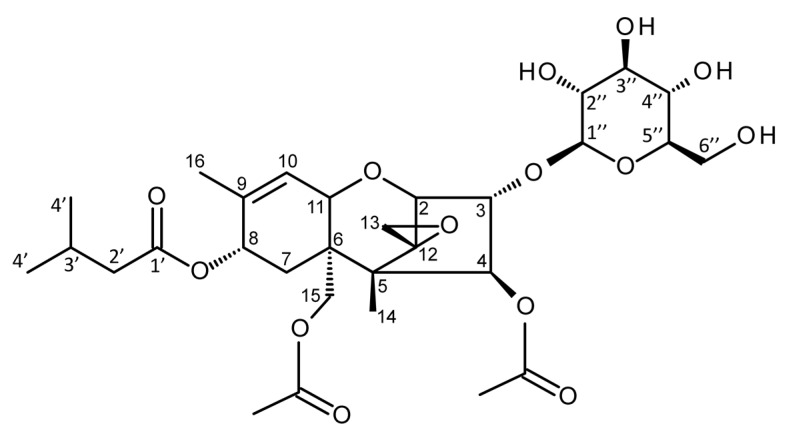
Structure of T-2 toxin-3-*O*-β-d-glucoside. The carbon atoms are numbered as they appear in Table 4 and Table 5.

The structures of all glucosides were elucidated by NMR (see Table 4 and Table 5; for numbering of C-atoms, see Figure 2). In each case, glucose was conjugated to C-3 (-3-Glc) of the trichothecene backbone. Furthermore, β-configuration was confirmed by coupling constants around 7.8 Hz for the anomeric proton (1″). The obtained T2-3-Glc spectra are identical with the previously published one [50]. 

When NMR analysis of HT2-Glc was performed for the first time, both the acetyl- and isovaleryl-group were cleaved off during NMR, most likely due to the extended period in methanol at ambient temperature. Consequently, instead of HT2-Glc, the structure of T2 tetraol-3-Glc was confirmed by NMR. Therefore, a second batch of HT2-Glc was produced and measured, this time in acetone-d_6_. Under these conditions, HT2-Glc was stable, and the structure was confirmed as HT2-3-Glc. Later on, we tested whether T2 tetraol-Glc can also be obtained under “standardized” conditions. Just a few drops of sodium-methoxide solution (25% *w*/*w* in methanol) into a HT2-3-Glc solution are enough to cleave off both functional groups. After 15 min, more than 50% of the initial HT2-3-Glc was converted, and both T2 triol-Glc and T2 tetraol-Glc were present in the sample. After 30 min, only trace amounts of HT2-3-Glc and T2 triol-3-Glc were left, and solely T2 tetraol-3-Glc was obtained. 

Also, in case of FUSX-Glc, stability problems during NMR were observed. The C-4 acetyl-group of FUSX-3-Glc was partially cleaved off, and NIV-3-Glc was obtained, whose structure was previously described [31,38]. Structural confirmation was still possible in the mixture. In case of 15-Ac-DON-Glc, this was not possible because already during the preparative purification step degradation to DON-3-Glc was observed as reported before [13]. In our experience, already 15-Ac-DON is unstable in aqueous methanol over time; it seems that the attachment of glucose makes the C-15 acetyl-group even more vulnerable. 15-Ac-DON-Glc could therefore only be confirmed by LC-MS/HRMS measurements and indirectly by analyzing the resulting DON-3-Glc. As so far only β-d-configured glucosides were obtained by OsUGT79, it reasonable to assume a conjugation of the glucose at C-3 in β-d-configuration also for 15-Ac-DON-Glc. 

## 3. Conclusions

In this paper we report the synthesis, purification, and characterization of β-glucoside-standards of several trichothecene toxins. Except for T2-3-β-glucoside, which was chemically synthesized before [50], all these standards can be regarded as novel. NMR analysis confirmed that in all cases glucosylation occurred at position C-3-OH by formation of an *O*-β-d-glycosidic link. Since we used defense-related crop-plant UDP-glucosyltransferases for synthesis, it appears likely that the obtained compounds are also relevant in nature. Enzymatic synthesis also allows a relatively convenient production of larger amounts of these glucosides, which could be useful in case toxicological studies with animals are of interest. 

Biochemical analysis of three trichothecene-conjugating UGTs indicated that the type A trichothecenes HT2 and T2 triol are the kinetically preferred substrates of Bradi5g03300 and HvUGT13248. HvUGT13248 shows the highest plasticity and also accepts C-4 acetylated trichothecenes to some degree. Nevertheless, it is obvious that C-4 acetylated trichothecenes are generally recalcitrant to glucosylation at C-3-OH. This is due to steric hindrance at the active site, as recently shown in the case of OsUGT79 [45]. By increasing the volume of the active site through mutagenesis, the activity of OsUGT79 could be expanded to include also T2, FUSX, and DAS. However, whether -3-*O*-glucosylation of C-4 acetylated trichothecenes is relevant in plant detoxification still needs to be shown, due to seemingly extensive deacetylation in various host plants. Since corresponding -3-*O*-β-glucoside standards are now available, it will be possible to determine their occurrences in natural samples and to have a closer look at the metabolism of such trichothecenes by plant detoxification systems. We have previously used some of these standards in other studies; for example, the occurrence of HT2-3-Glc (also NIV-3-Glc) in Finnish cereals could so be documented for the first time [30]. Using the here described HT2-3-Glc standard, a series of studies investigating the metabolism of T2/HT2 in barley, wheat, and oats [51,52,53] showed that HT2-3-Glc is the major detoxification product of both T2 and HT2. 

## 4. Materials and Methods

### 4.1. Materials

Uridine 5′-diphosphoglucose disodium salt hydrate (UDP-glucose) from *Saccharomyces cerevisiae* (cat. no. U4625) was purchased from Sigma-Aldrich (Vienna, Austria). *Escherichia coli* Rosetta™ (DE3) was purchased from Novagen (Madison, WI, USA), and *E. coli* SHuffle^®^ T7 Express *lysY* was purchased from New England Biolabs (Frankfurt am Main, Germany). DON, T2, HT2, 15-Ac-DON, DAS, NEO, FUSX, T2 triol, and T2 tetraol were purified at Romer Labs (Tulln, Austria). 4-deoxy-T2 [54] was provided by Susan McCormick from the Agricultural Research Service, U.S. Department of Agriculture (Peoria, IL, USA). 

### 4.2. Expression and Purification of OsUGT79, HvUGT13248, and Bradi5g03300

OsUGT79 (GenBank NM_001058779.1/BAF14158.1), Bradi5g03300 (GenBank KQJ81826.1), and HvUGT13248 (GenBank ADC92550.1) were expressed in *E. coli* using the sequences optimized for expression in *S. cerevisiae* as previously reported [37,41]. As previously described [44], OsUGT79 was expressed as a fusion protein with *N*-terminal polyhistidine-tag and maltose binding protein (malE) (*n*His_6_-MalE-OsUGT79). 

Bradi5g03300 was expressed similarly but with *C*-terminal histidine tag (MalE-Bradi5g03300-*c*His_6_). The gene was amplified with the oligonucleotide primers 5′-AAAcatatgGACTCTACTGGTAAATCCG-3′ and 5′-TTTaagcttAGAAGAGGAATATTTGGCAGC-3′, and cloned into expression vector pCA02b (Supplemental Figure S4) using the restriction sites *Nde*I and *Hin*dIII. 

HvUGT13248 was expressed with *C*-terminal His-tag (HvUGT13248-*c*His_6_) in pET21a (Novagen, Madison, WI, USA) and as MalE-HvUGT13248-*c*His_6_ in pCA02b. The HvUGT13248 gene was amplified with primers 5′-TATgctagcATGGAGACCACGGTCAC-3′ and 5′-ATTaagcttTATTGACGAATACTTGGTAG-3′, and ligated to pET21a/pCA02b using the restriction sites *Nhe*I and *Hind*III. *n*His_6_-MalE-OsUGT79 was expressed in *E. coli* Rosetta™ (DE3), MalE-Bradi5g03300-*c*His_6_, and HvUGT13248-*c*His_6_ in *E. coli* SHuffle^®^ T7 Express *lysY*.

Protein expression was carried out in modified terrific broth (10 g/L tryptone, 20 g/L yeast extract, 5 g/L glycerol, and 100 mM potassium phosphate buffer pH 7) with 100 mg/L ampicillin, supplemented with 35 mg/L chloramphenicol when *E. coli* Rosetta was used. Isopropyl-β-d-1-thiogalactopyranoside (IPTG) was added to a final concentration of 1 mM when the optical density (OD_600_) reached 0.5–1. The flasks were further incubated for 16 h at 20 °C and 100 rpm. After that period, the biomass was harvested by centrifugation (4000× *g*, 15 min) and re-suspended in 50 mM potassium phosphate buffer pH 7 with 10% (*w*/*v* glycerol) and 1% (*w*/*v*) Triton X-100. The cells were disrupted by sonication with a Branson Sonifier W-250 D (Branson Ultrasonics Corporation, Danbury, CT, USA). The cell extract was cleared by centrifugation at 30,000× *g*. Protein purification was performed by immobilized metal affinity chromatography (IMAC) on Ni^2+^-charged HisTrap Crude FF columns, 1 mL or 5 mL (GE Healthcare, Chalfont St Giles, Great Britain, UK). After the cell extract was loaded onto the column, it was washed with a 50 mM potassium phosphate buffer pH 7 containing 500 mM NaCl and 25 mM imidazole. Bound protein was eluted with the same buffer containing 500 mM imidazole. After IMAC purification, the protein-containing fractions were pooled, and the buffer was changed to 50 mM potassium phosphate pH 7 + 50 mM KCl + 10% (*w*/*v*) glycerol by gel filtration with Sephadex G25 (GE Healthcare) or by using Amicon Ultra-15 Centrifugal Filter units with a molecular weight cut-off of 30 kDa (Merck Millipore Ltd., Darmstadt, Germany), which were also used for concentrating the protein if necessary. Enzyme preparations were stored at −80 °C.

### 4.3. Glycosyltransferase Assays

Glucosyltransferase activity was determined with the UDP-Glo assay from Promega (Fitchburg, WI, USA). All assays were performed at 25 °C in 100 mM potassium phosphate buffer at pH 7 and contained 1 mM UDP-glucose and 25 µM of the aglycone. The amount of added glucosyltransferase was adjusted so that the signal was within the calibration range. Blanks contained H_2_O instead of the toxin-solution. A calibration curve ranging from 0.2 to 25 µM UDP was generated. After 10–15 min of reaction, an equal volume of the UDP detection reagent was added and incubated for 60 min (25 °C). Luminescence was measured on an EnSpire 2300 Multilabel Reader (PerkinElmer, Waltham, MA, USA). All assays were performed in quadruplicate.

Enzyme kinetics with DON, NIV, and HT2 were determined with the assay described above, except that substrate concentrations varied from about 5 µM up to 25 mM (DON), 8 mM (NIV), or 10 mM (HT2). Kinetic parameters (*K*_M_, *V*_max_) were calculated as average from at least three independent measurements. Displayed activity units (μmol min^−1^ mg^−1^) refer to the release of UDP per mg of protein. Data analysis was performed with SigmaPlot 11.0 (Systat Software, San Jose, CA, USA). 

### 4.4. LC-MS/MS Measurements

LC-MS/MS measurements were performed on a QTrap 4000 (Sciex, Foster City, CA, USA) equipped with a TurboV electrospray ionization source and coupled to a 1290 ultra-high performance liquid chromatography (UHPLC) system (Agilent Technologies, Waldbronn, Germany). A generic water-methanol gradient with 5 mM ammonium acetate as modifier in both eluents was applied for chromatographic separation. Either a Gemini C-18 column (150 × 4.6 mm, 5 µm, Phenomenex, Aschaffenburg, Germany) or a Kinetex C-18 (150 × 2.1 mm, 2.6 µm, Phenomenex) column was used. The selected reaction monitoring transitions of the aglycons and the later optimized glucosides are provided in Table 6. Theoretical selected reaction monitoring transitions from the *m*/*z*-values of the trichothecene-glucosides to the aglycons were applied before standards became available. The instrument was operated and data were evaluated with Analyst 1.6.2 (Sciex, Foster City, CA, USA). Alternatively, in some cases the LC-HRMS method described below for the characterization of the glucosides was also used in the screening process. 

### 4.5. Large Scale Glucosylation of Trichothecenes and Purification Thereof

Table 3 provides an overview of the glucosylation batches and the individual conditions applied. 

The purification of the glucosides was performed on an 1100 series preparative HPLC system (Agilent Technologies) equipped with an evaporative light scattering detector (ELSD; Sedex 85, Sedere LT-ELSD, Altfortville Cedex, France). The eluents were pure water (A) and methanol (B), and the column and specific purification conditions for the individual glucosides are specified below. After purification, all relevant fractions were pooled, evaporated on a rotary evaporator at 35 °C, and finally lyophilized to obtain the glucosides in a crystalline form.

For the ELSD-guided purification of HT2-Glc, 15-Ac-DON-Glc, and T2 triol-Glc, a Gemini NX column (150 × 21.2 mm, 5 µm, Phenomenex, Aschaffenburg, Germany) and a flow rate of 20 mL/min, 20 mL/min, and 16 mL/min, respectively, were applied. The gradient for HT2-Glc (7.4 min) was as follows: 0–2 min: 40% B, 2–10 min: linear gradient from 40 to 100% B, 10–11 min: 100% B, followed by a column re-equilibration. HT2-Glc was collected by ELSD-peak-based fractionation around 7.4 min. For T2 triol-Glc (6.6 min), the gradient was adapted to: 0–3 min: 50% B; 3–7.5 min gradient from 50 to 80% B; 7.6–10 min: 100% B, 10.1 min 50% B. In case of 15-Ac-DON-Glc, the following gradient was applied: 0–2 min: 20% B, 2–10 min: linear gradient from 20 to 100% B, 10–11 min: 100% B, followed by a column re-equilibration. Two peaks were collected (4.0 min and 6.6 min) containing DON-3-Glc and a 15-Ac-DON-Glc. The latter partially degraded during the evaporation and lyophilization process to DON-3-Glc. 

FUSX-Glc and T2-Glc were purified on a SunFire Prep C18 OBD column (19 × 100 mm, 5 µm, Waters, Milford, MA, USA) using flow rates of 16 mL/min and 21 mL/min. Due to the UV-activity of FUSX-Glc (4.4 min), a UV-based fractionation at 220 nm using the following gradient was performed: 0–1 min: 20% B, 1–6 min: gradient from 20% B to 80% B; 6.1–8.5 min: 100% B and column equilibration. T2-Glc (5.3 min) was purified based on the ELSD-signal applying the following gradient: 0–1 min 40% B; 1–5 min linear gradient from 40 to 80% B; 5–7.5 min increase to 100% B, before the column was flushed and re-equilibrated. 

For the purification of DAS-Glc an XBridge C18 OBD column (19 × 100 mm, 5 µm, Waters, Milford, MA, USA) with a flow rate of 21 mL/min was used. The gradient started with a 1 min holding time at 30% B followed by a linear increase to 100% B within the next 7 min; thereafter, the column was flushed with 100% B for 1.5 min and re-equilibrated with the starting conditions. DAS-Glc was collected by an ELSD-peak based fractionation around 4.8 min. A purity check by LC-MS/MS showed that the purified samples still contained around 5% DAS. Therefore, another purification step using the Gemini NX column (150 × 21.2 mm, 5 µm), and a flow rate of 21 mL/min was applied: 0–1 min: 30% B, 1–8 min: 30→100% B, 8–9.5 min: 100% B, followed by a column re-equilibration. DAS-Glc was collected by ELSD-peak-based fractionation around 4.8 min. 

NEO-Glc was purificed on an XBridge C18 OBD column (19 × 100 mm, 5 µm) using a flow rate of 21 mL/min. The starting conditions of 10% B were hold for 1 min, and a linear gradient to 100% was applied within the next 7 min. Thereafter, the column was flushed for 1.5 min, followed by a re-equilibration step. NEO-Glc was collected based on the ELSD-peak using a peak-based fractionation around 4.3 min. A purity check by LC-MS/MS revealed two peaks with the mass of NEO-Glc, and therefore another purification step was applied, this time using a Gemini NX (150 × 21.2 mm, 5 µm) and a flow rate of 21 mL/min. After a hold time of 1 min at 10% B, a linear gradient to 100% B was applied within the next 7 min, followed by 1.5 min column flushing at 100% B and column re-equilibration at the starting conditions. The first eluting, higher intensive peak of NEO-Glc was collected based on the ELSD-peak around 5.5 min. The second minor peak was collected via time-based fractionation between 5.65 and 5.75 min. 

### 4.6. Characterization of the Produced Glucosides

LC-HRMS and LC-MS/HRMS were performed on a 1290 UHPLC system coupled to a 6550 iFunnel quadrupole time-of-flight (QTOF) LC-MS instrument (all Agilent Technologies). For chromatographic separation, a Zorbax SB C18 column (150 × 2.1 mm, 1.8 µm, Agilent Technologies) at 30 °C and a flow rate of 250 µL/min were used. The eluents were composed of water (A) and methanol (B) and contained both 0.1% formic acid and 1 mM ammonium formate, and the applied gradient was as follows: 0–0.5 min: 10% B; 0.5–10 min: linear gradient from 10% B to 100% B, 10–12 min: column flushing with 100% B, 12.1–15 min: column equilibration with 10% B. The MS parameters were as follows: gas temperature 130 °C, drying gas flow 14 L/min, nebulizer 30 psig, sheath gas temperature 300 °C and flow 10 L/min, capillary voltage 4000 V and nozzle voltage 500 V. MS full scan (*m*/*z* 100 to 1000) and targeted product ions scans (*m*/*z* 50 to 1000) were performed at a scan rate of 3 spectra/s. In positive ionization mode, the funnel parameters were set to 150 V and 100 V for delta V1 and delta V2 (in negative mode these were of the opposite charge), and to 150 V and 60 V for the high pressure and low pressure funnel, respectively. The precursor ions were isolated using an isolation width of 1.3 *m*/*z*, and different collision energies were applied. Prior to use, the QTOF was tuned and calibrated, and during the run two references masses (*m*/*z* 121.0508 and *m*/*z* 922.0098 in positive ionization mode and *m*/*z* 112.9855 and *m*/*z* 966.0007 in negative ionization mode) were constantly infused via a second nebulizer to ensure mass accuracy. MassHunter Workstation LC/MS Data Acquisition version B.08.00 SP1 and MassHunter Workstation Qualitative Analysis version B.07.00 SP2 were used for data acquisition, handling, and evaluation.

NMR spectra were obtained on a Bruker Avance DRX-400 MHz spectrometer (T2 tetraol-3-Glc, and HT2-3-Glc; operating at 400 MHz for ^1^H and 100 MHz for ^13^C) or a Bruker Avance III HD 600 FT-NMR spectrometer (all other glucosides operating at 600 MHz for ^1^H and 151 MHz for ^13^C using a Cryoprobe Prodigy™ probehead). The samples were dissolved in acetone-d_6_ (HT2-3-Glc and T2-triol-3-Glc), acetonitrile-d_3_ (FUSX-3-Glc), or methanol-d_4_ (all others) and measured at ambient temperature. Deuterated solvents were purchased from Eurisotop (Gif sur Yvette Cedex, Paris, France). All chemical shifts are provided in ppm relative to tetramethylsilane, and calibration was performed on the basis of residual solvent resonances (acetone-d_6_ 2.05 ppm for ^1^H, 29.92 ppm for ^13^C; acetonitrile-d_3_ 1.94 for ^1^H, 1.39 ppm for ^13^C; methanol-d_4_ 3.31 ppm for ^1^H, 49.15 ppm for ^13^C). All pulse programs were taken from the Bruker software library. The NMR data were evaluated using TOPSPIN 1.3 and TopSpin 3.5 (Bruker BioSpin GmbH, Billerica, MA, USA). Structure elucidation and signal assignment were carried out based on 1D (^1^H, ^13^C) and 2D (^1^H^1^H correlation spectroscopy, ^1^H^13^C hetero-nuclear single quantum correlation, and ^1^H^13^C hetero-nuclear multiple bond correlation) NMR spectra. The only exception was T2-3-Glc, for which only 1D-spectra were acquired, which were compared with the already published spectra [50]. 

## Figures and Tables

**Table 1:** Relative activities of UDP-glucosyltransferases with different substrates (25 µM in assay) determined at pH 7 (100 mM potassium phosphate) and 25 °C with 1 mM UDP-glucose. Released UDP was detected with the UDP-Glo Assay (Promega). The values shown are the activities relative to deoxynivalenol (DON, 100%). The results are the means of quadruplicate determination ± standard deviations.

Substrate	Relative Activity (%)		
	OsUGT79	HvUGT13248	Bradi5g03300
Deoxynivalenol	100	100	100
15-Acetyl-deoxynivalenol	31 ± 3	424 ± 28	21 ± 5
Nivalenol	64 ± 3	360 ± 26	41 ± 6
Fusarenon X	4.6 ± 2.2	29 ± 3	nd
T-2 toxin	nd	21 ± 12	nd
HT-2 toxin	97 ± 16	1634 ± 177	1065 ± 68
T-2 triol	117 ± 21	1646 ± 124	1391 ± 69
T-2 tetraol	110 ± 16	382 ± 41	195 ± 6
4-Deoxy T-2 toxin	63 ± 3	204 ± 6	169 ± 16
Neosolaniol	nd	19 ± 9	nd
4,15-Diacetoxyscirpenol	nd	10 ± 3	nd
Zearalenone	89 ± 2	153 ± 4	20 ± 4
Kaempferol	nd	22 ± 3	nd
Quercetin	nd	7 ± 3	nd

nd, not detectable = not significantly different from control (95% level, *p* > 0.05).

**Table 2:** Kinetic analysis of HvUGT13248 and Bradi5g03300 determined at pH 7 (100 mM potassium phosphate) and 25 °C with 1 mM UDP-glucose. Released UDP was detected with the UDP-Glo Assay (Promega). The results are the means of three independent determinations ± standard deviations. Since the enzyme preparations were not pure, *V*_max_/*K*_M_ was calculated instead of *k*_cat_/*K*_M_.

Enzyme	Substrate	*K*_M_ (mM)	*V*_max_ (µmol min^−1^ mg^−1^)	*V*_max_/*K*_M_
HvUGT13248	Deoxynivalenol	1.5 ± 0.3	0.22 ± 0.03	0.14
	Nivalenol	0.64 ± 0.12	0.47 ± 0.05	0.73
	HT-2 toxin	0.053 ± 0.021	0.21 ± 0.02	4.0
Bradi5g03300	Deoxynivalenol	0.37 ± 0.05	0.019 ± 0.001	0.05
	Nivalenol	1.3 ± 0.3	0.014 ± 0.001	0.01
	HT-2 toxin	0.12 ± 0.02	0.042 ± 0.004	0.35

**Table 3:** Overview of conditions and yields of different glucosylation batches. 15-Ac-DON, 15-acetyl-deoxynivalenol; HT2, HT-2 toxin; T2 triol, T-2 triol; FUSX, fusarenon X; T2, T-2 toxin, DAS, 4,15-diacetoxyscirpenol; NEO, neosolaniol; Tris, tris(hydroxymethyl)aminomethane buffer; KPP, potassium phosphate buffer; UGT, UDP-glucosyltransferase.

	15-Ac-DON	HT2	HT2	T2 triol	FUSX	T2	DAS	NEO
Total amount of toxin (mg)	10	5	5	2.2	6	5	5	6
Concentration toxin (mM)	1.5	0.6	0.6	1.15	2.1	2.8	2.7	2.8
UDP-glucose (mM)	2.3	1	1.2	10	10	10	10	10
Buffer (100 mM, pH 7)	Tris	Tris	Tris	Tris	KPP	KPP	KPP	KPP
Temperature (°C)	25	25	25	37	25	25	25	25
Reaction time (h)	24	24	4	16	72	72	72	72
UGT (mg/mL)	1 (A)	1 (A)	1 (A)	2 (A)	1.7 (B)	1.7 (B)	3.2 (B)	4 (B)
Sucrose (mM)					50	50	50	50
Sucrose synthase (mg/mL)					0.9	0.9	1.8	1.8
Final amount glucoside (mg)	not stable	5.6 (C)	7.0	1.9	2.2	3.4	5.9	1.5/0.4 (D)

(A)—OsUGT79, (B)—HvUGT13248, (C)—Degraded to T-2 tetraol-3-*O*-β-d-glucoside during NMR, (D)—Neosolaniol-3-*O*-β-d-glucoside/iso-neosolaniol-3-*O*-β-d-glucoside.

**Table 4:** ^1^H NMR data (δ, ppm; multiplicity; J, Hz; number of H-atoms).

Pos.	T-2 Toxin-3-*O*-β-d-glucoside in Methanol-d_4_ (A)	HT-2 Toxin-3-*O*-β-d-glucoside in Acetone-d_6_	T-2 Triol-3-*O*-β-d-glucoside in Acetone-d_6_	T-2 Tetraol-3-*O*-β-d-glucoside in Methanol-d_4_	4,15-Diacetoxyscirpenol-3-*O*-β-d-glucoside in Methanol-d_4_	Neosolaniol-3-*O*-β-d-glucoside in Methanol-d_4_	Iso-neosolaniol-3-*O*-β-d-glucoside in Methanol-d_4_	Fusarenon X-3-*O*-β-d-glucoside in Acetonitrile-d_3_
2	3.72 (d, 5.0, 1H)	3.54 (d, 4.7, 1H)	3.48 (d, 4.9, 1H)	3.57 (d, 4.8, 1H)	3.70 (d, 4.8, 1H)	3.69 (d, 5.0, 1H)	3.68 (d, 5.0, 1H)	3.81 (d, 4.8, 1H)
3	4.48 (dd, 5.0, 3.1, 1H)	4.31 (b, 1H)	4.24 (dd, 4.9, 3.2, 1H)	4.23 (dd, 4.8, 3.3, 1H)	4.44 (dd, 4.8, 3.3 1H)	4.46 (m, 1H)	4.38 (dd, 5.0, 3.2, 1H)	4.42 (dd, 4.8, 3.5, 1H)
4	5.98 (d, 3.1, 1H)	4.60 (b, 1H)	4.83 (m, 1H)	4.36 (d, 3.3, 1H)	5.79 (d, 3.2, 1H)	6.06 (d, 3.0, 1H)	6.09 (d, 3.2, 1H)	5.80 (d, 3.5, 1H)
4-Ac	2.09 (s, 3H)	-	-	-	2.10 (s, 3H)	2.09 (s, 3H)	2.10 (s, 3H) **(D)**	2.09 (s, 3H)
7	2.38 (dd, 15.2, 5.8, 1H) 1.94 (bd, 15.2, 1H)	2.35 (dd, 15.1, 5.6, 1H) 2.01 (bd, 15.0, 1H)	2.26 (dd, 15.0, 5.8, 1H) 1.86 (d, 15.0, 1H)	2.17 (dd, 14.3, 5.1, 1H) 1.99 (bd, 14.3, 1H)	2.01 (m, 1H) 1.81 (m, 1H)	2.25 (dd, 14.5, 5.6, 1H) 1.85 (bd, 14.5, 1H)	2.24 (dd, 15.1, 5.9, 1H) 1.92 (bd, 15.1, 1H)	4.79 (s, 1H)
8	5.33 (d, 5.7, 1H)	5.28 (d, 5.5, 1H)	5.26 (d, 5.8, 1H)	4.02 (bd, 4.9, 1H)	2.05–1.90 (m, 2H)	4.06 (d, 5.5, 1H)	5.24 (d, 5.7, 1H)	-
8-Ac	-	-	-	-	-	-	2.05 (s, 3H) **(D)**	-
10	5.75 (d, 5.9, 1H)	5.69 (d, 5.5, 1H)	5.66 (m, 1H)	5.55 (bd, 5.6, 1H)	5.48 (d, 5.5, H)	5.60 (d, 5.8, 1H)	5.77 (d, 6.0, 1H)	6.58 (dd, 5.9, 1.5, 1H)
11	4.38 (m, 1H)	4.20 (d, 5.5, 1H)	4.18 (d, 5.9, 1H)	3.87 (bd, 5.6, 1H)	4.15 (d, 5.5, 1H)	4.38 (d, 5.8, 1H)	4.31 (d, 6.0, 1H)	4.67 (d, 5.9, 1H)
13	3.04 (d, 3.8, 1H) 2.87 (d, 3.8, 1H)	2.95 (d, 4.0, 1H) 2.80 (d, 4.0, 1H)	2.89 (d, 4.2, 1H) 2.75 (d, 4.2, 1H)	2.93 (d, 4.1, 1H) 2.80 (d, 4.1, 1H)	3.02 (d, 3.9, 1H) 2.85 (d, 3.9, 1H)	3.03 (d, 4.0, 1H) 2.85 (d, 4.0, 1H)	3.02 (d, 4.0, 1H) 2.85 (d, 4.0, 1H)	3.05 (d, 4.2, 1H) 2.98 (d, 4.2, 1H)
14	0.74 (s, 3H)	0.83 (s, 3H)	0.84 (s, 3H)	0.86 (s, 3H)	0.75 (s, 3H)	0.78 (s, 3H)	0.79 (s, 3H)	0.94 (s, 3H)
15	4.38 (d, 12.6, 1H) 4.09 (d, 12.6, 1H)	4.24 (d, 12.2, 1H) 3.95 (d, 12.2, 1H)	3.84 (d, 12.1, 1H) 3.53 (d, 12.0, 1H)	3.74 (d, 12.4, 1H) 3.41 (d, 12.4, 1H)	4.31 (d, 12.3, 1H) 4.04 (d, 12.3, 1H)	4.39 (d, 12.5, 1H) 4.20 (d, 12.5, 1H)	3.97 (d, 12.2, 1H) 3.56 (d, 12.2, 1H)	3.89 (d, 12.3, 1H) 3.66 (d, 12.3, 1H)
15-Ac	2.06 (s, 3H)	2.03 (s, 3H)	-	-	2.05 (s, 3H)	2.07 (s, 3H)	-	-
16	1.75 (s, 3H)	1.72 (s, 3H)	1.70 (s, 3H)	1.83 (s, 3H)	1.73 (s, 3H)	1.84 (s, 3H)	1.76 (s, 3H)	1.82 (s, 3H)
2′	2.15 (m, 2H)	2.15 (d, 7.0, 2H)	2.21 (d, 6.9, 2H)	-	-	-	-	-
3′	ca. 2.06 (m, 1H)	ca. 2.05 (1H) **(B)**	2.06 (m, 1H)	-	-	-	-	-
4′	0.97 (d, 6.6, 3H) 0.96 (d, 6.6, 3H)	0.97 (d, 6.4, 3H) 0.96 (d, 6.4, 3H)	0.95 (d, 6.7, 3H) 0.94 (d, 6.7, 3H)	-	-	-	-	-
1″	4.44 (d, 7.8, 1H)	4.72 (d, 7.9, 1H)	4.66 (d, 7.9, 1H)	4.57 (d, 7.9, 1H)	4.42 (d, 7.9, 1H)	4.45 (d, 7.8, 1H)	4.45 (d, 7.8, 1H)	4.39 (d, 7.8, 1H)
2″	3.21 (m, 1H)	3.29 (t, 8.2, 1H)	3.26 (dd, 8.6, 7.9, 1H)	3.25 (b, 1H)	3.24 (dd, 8.9, 7.9, 1H)	3.25 (dd, 9.0, 7.9, 1H)	3.25 (m, 1H)	3.16 (m, 1H)
3″	3.35 (t, 9.0, 1H)	3.43 (t, 8.8, 1H)	3.42 (t, 8.7, 1H)	3.37 (bt, 7.2, 2H) **(C)**	3.35 (t, 8.9, 1H)	3.35 (t, 9.0, 1H)	3.35 (m, 1H)	3.30 (m, 1H)
4″	ca. 3.26 (m, 1H)	3.40 (t, 9.1, 1H)	3.37 (dd, 9.3, 8.7, 1H)	3.37 (bt, 7.2, 2H) **(C)**	3.31 (1H) **(B)**	3.30 (t, 9.0, 1H)	3.30 (m, 1H)	3.25 (m, 1H)
5″	3.21 (ddd, 9.7, 5.7, 2.2, 1H)	3.34 (b, 1H)	3.32 (dd, 9.4, 5.4, 1H)	3.27 (b, 1H)	3.21 (ddd, 9.5, 5.6, 2.2, 1H)	3.21 (ddd, 9.5, 5.7, 2.2, 1H)	3.22 (m, 1H)	3.25 (m, 1H)
6″	3.83 (dd, 12.1, 2.2, 1H) 3.65 (dd, 12.1, 5.7, 1H)	3.84 (bd, 10.6, 1H) 3.70 (m, 1H)	3.82 (m, 1H) 3.66 (m, 1H)	3.83 (dd, 12.1, 2.0, 1H) 3.68 (dd, 12.1, 5.0, 1H)	3.83 (dd,12.0, 2.3, 1H) 3.65 (dd, 12.0, 5.6, 1H)	3.83 (dd, 12.0, 2.2, 1H) 3.65 (dd, 12.0, 5.7, 1H)	3.82 (dd, 12.2, 2.4, 1H) 3.65 (m, 1H)	3.6 (m, 2H)

Ac, acetyl-group; Multiplicities are abbreviated as s (singlet), d (doublet), bd (broad doublet), bt (broad triplet), dd (doublet of doublets), t (triplet), m (multiplet), and b (broad signal). **(A)**—Only ^1^H- and ^13^C-spectra were acquired and compared to the already published spectra acquired on an Agilent 600 MHz NMR-spectrometer [50]. **(B)**—Overlapping with solvent signal. **(C)**—In this case it was not possible to assign the specific position of the two protons with the chosen measurement conditions. **(D)**—Indicates that shifts for positions 4-Ac and 8-Ac may be reversed.

**Table 5:** ^13^C NMR data (δ, ppm).

Pos.	T-2 Toxin-3-*O*-β-d-glucoside in Methanol-d_4_ (A)	HT-2 Toxin-3-*O*-β-d-glucoside in Acetone-d_6_	T-2 Triol-3-*O*-β-d-glucoside in Acetone-d_6_	T-2 Tetraol-3-*O*-β-d-glucoside in Methanol-d_4_	4,15-Diacetoxyscirpenol-3-*O*-β-d-glucoside in Methanol-d_4_	Neosolaniol-3-*O*-β-d-glucoside in Methanol-d_4_	Iso-neosolaniol-3-*O*-β-d-glucoside in Methanol-d_4_	Fusarenon X-3-*O*-β-d-glucoside in Acetonitrile-d_3_
2	80.6	78.8	79.9	80.4 **(B)**	80.8	80.6	80.7	80.7
3	84.0	85.3	86.7	87.7	84.4	84.1	84.8	83.3
4	81.3	78.8	79.7	80.5 **(B)**	81.8	81.3	81.8	80.9
4-Ac	172.3 & 20.9	-	-	-	172.5 & 21.0	172.3 & 21.0	172.6 & 21.0	172.0 & 21.2
5	50.2	48.6	49.4	50.2	50.4	50.2	49.9	50.1
6	44.5	42.6	45.1	46.2	45.6	44.9	45.9	54.4
7	28.9	27.2	28.3	30.0	22.3	31.8	28.4	74.8
8	69.5	68.0	69.2	67.0	29.0	67.2	70.1	200.4
8-Ac	-	-	-	-	-	-	172.6 & 21.3	-
9	137.5	134.8	135.6	141.5	141.9	141.6	137.6	136.7
10	125.2	124.8	126.3	122.6	119.8	121.9	125.4	138.9
11	68.6	67.0	68.2	70.2	69.5	69.1	69.2	70.5
12	65.4	64.2	65.5	65.7	65.4	65.6	65.7	65.4
13	48.0	45.9	46.9	47.5	47.9	48.1	48.1	47.0
14	7.2	6.6	7.4	7.4	7.2	7.2	7.2	8.0
15	65.9	64.2	63.5	63.2	64.8	66.2	63.7	61.4
15-Ac	172.4 & 21.4	169.6 & 20.3	-	-	172.6 & 21.1	172.8 & 21.3	-	-
16	20.6	19.5	20.4	20.9	23.4	21.0	20.5	15.4
1′	174.1	171.7	172.7	-	-	-	-	-
2′	44.7	43.1	44.1	-	-	-	-	-
3′	27.1	25.5	26.4	-	-	-	-	-
4′	22.9	21.8 & 21.7	22.7	-	-	-	-	-
1″	103.9	102.4	103.5	104.4	104.3	103.9	104.3	103.3
2″	74.9	73.6	74.6	75.0	74.9	74.9	75.1	74.4
3″	78.2	77.1	78.1	78.3 **(C)**	78.2	78.2	78.1	77.5
4″	71.6	70.7	71.7	71.4	71.5	71.5	71.5	71.4
5″	78.4	76.8	77.7	78.3 **(C)**	78.4	78.4	78.4	77.5
6″	62.8	61.9	62.9	62.6	62.7	62.7	62.7	62.7

Ac, acetyl-group. **(A)**—Only ^1^H- and ^13^C-spectra were acquired and compared to the already published spectra acquired on an Agilent 600 MHz NMR-spectrometer [50]. **(B)**—Indicates that shifts for positions 2 and 4 may be reversed. **(C)**—Indicates that shifts for positions 3″ and 5″ may be reversed.

**Table 6:** Selected reaction monitoring transitions of the investigated aglycons and glucosides in positive or negative electrospray ionization mode.

Compound Name	Q1 (*m*/*z*) and Ion Species	DP (V)	Q3 (*m*/*z*)	CE (eV)
15-acetyl-deoxynivalenol	339.1 [M + H]^+^	86	261.0/321.0	15/11
15-acetyl-deoxynivalenol-glucoside	518.0 [M + NH_4_]^+^	56	339.0/321.1	19/27
4,15-diacetoxyscirpenol	384.2 [M + NH_4_]^+^	51	307.2/105.1	17/61
4,15-diacetoxyscirpenol-3-*O*-β-d-glucoside	546.0 [M + NH_4_]^+^	66	307.2/105.0	21/83
fusarenon X	413.3 [M + CH_3_COO]^−^	−40	59.1/262.9	−44/−22
fusarenon X-3-*O*-β-d-glucoside	561.1 [M + CH_3_COO]^−^	−80	515.1/244.8	−22/−34
HT-2 toxin	442.2 [M + NH_4_]^+^	70	215.1/197.1	19/25
HT-2 toxin-3-*O*-β-d-glucoside	604.4 [M + NH_4_]^+^	51	263.3/215.1	27/25
neosolaniol	400.2 [M + NH_4_]^+^	46	185.0/215.0	25/29
neosolaniol-3-*O*-β-d-glucoside	562.2 [M + NH_4_]^+^	71	305.0/185.0	25/39
iso-neosolaniol-3-*O*-β-d-glucoside	562.2 [M + NH_4_]^+^	66	215.2/202.9	35/27
T-2 tetraol	316.2 [M + NH_4_]^+^	31	215.3/281.4	13/25
T-2 tetraol-3-*O*-β-d-glucoside	478.3 [M+NH_4_]^+^	46	215.1/233.2	21/15
T-2 triol	400.2 [M + NH_4_]^+^	41	215.2/281.3	17/13
T-2 triol-3-*O*-β-d-glucoside	562.3 [M + NH_4_]^+^	41	215.5/233.5	20/10
T-2 toxin	484.3 [M + NH_4_]^+^	56	215.2/185.1	29/31
T-2 toxin-3-*O*-β-d-glucoside	646.3 [M + NH_4_]^+^	66	305.0/215.0	30/35

Q1, precursor ion mass; DP, declustering potential; Q3, fragment ion masses; CE, collision energies.

## Supplementary Materials

The following are available online at http://www.mdpi.com/2072-6651/10/3/111/s1, Figure S1: High resolution product ion spectra of the ammonium adduct of T-2 toxin-glucoside, HT-2 toxin-glucoside, T-2 triol-glucoside, and T-2 tetraol-glucoside, Figure S2: High resolution product ion spectra of the ammonium adduct of neosolaniol-glucoside, iso-neosolaniol-glucoside, and 4,15-diacetoxyscirpenol-glucoside, Figure S3: High resolution product ion spectra of the ammonium adduct of 15-acetyl-deoxynivalenol-glucoside and fusarenon X-glucoside, Figure S4: Topology of pCA02a/b.

## Abbreviations

-Glc	-glucoside
-3-Glc	-3-*O*-β-d-glucoside
3-Ac-DON	3-acetyl-deoxynivalenol
15-Ac-DON	15-acetyl-deoxynivalenol
DAS	4,15-diacetoxyscirpenol
DON	deoxynivalenol
FHB	Fusarium head blight
FUSX	fusarenon X (4-acetyl-nivalenol)
HT2	HT-2 toxin
IMAC	immobilized metal affinity chromatography
IPTG	isopropyl-β-d-1-thiogalactopyranoside
iso-NEO	iso-neosolaniol (15-deacetyl-8-acetyl-neosolaniol)
MalE	maltose binding protein
NEO	neosolaniol
NIV	nivalenol
LC-MS/MS	liquid chromatography coupled to tandem mass spectrometry
LC-(MS/)HRMS	liquid chromatography coupled to (tandem) high resolution mass spectrometry
T2	T-2 toxin
T2 triol	T-2 triol
T2 tetraol	T-2 tetraol
QTL	quantitative trait locus
UGT	UDP-glucosyltransferase
UHPLC	ultra high performance liquid chromatography
