# Supplementary material for: UDP-Glucosyltransferases from Rice, Brachypodium, and Barley: Substrate Specificities and Synthesis of Type A and B Trichothecene-3-O-β-d-glucosides
Source: Toxins (Basel). 2018 Mar 6;10(3):111. doi: 10.3390/toxins10030111 (PMC5869399; doi:10.3390/toxins10030111)
Supplement: Supplementary file 1 [file toxins-10-00111-s001.pdf]

Supplementary Materials: UDP-Glucosyltransferases from Rice, *Brachypodium* and Barley: Substrate Specificities and Synthesis of Type A and B Trichothecene-3-*O*-β-D-glucosides

Herbert Michlmayr, Elisabeth Varga, Alexandra Malachová, Philipp Fruhmann, Marta Piątkowska, Christian Hametner, Jana Šofrová, Günther Jaunecker, Georg Häubl, Marc Lemmens, Franz Berthiller and Gerhard Adam

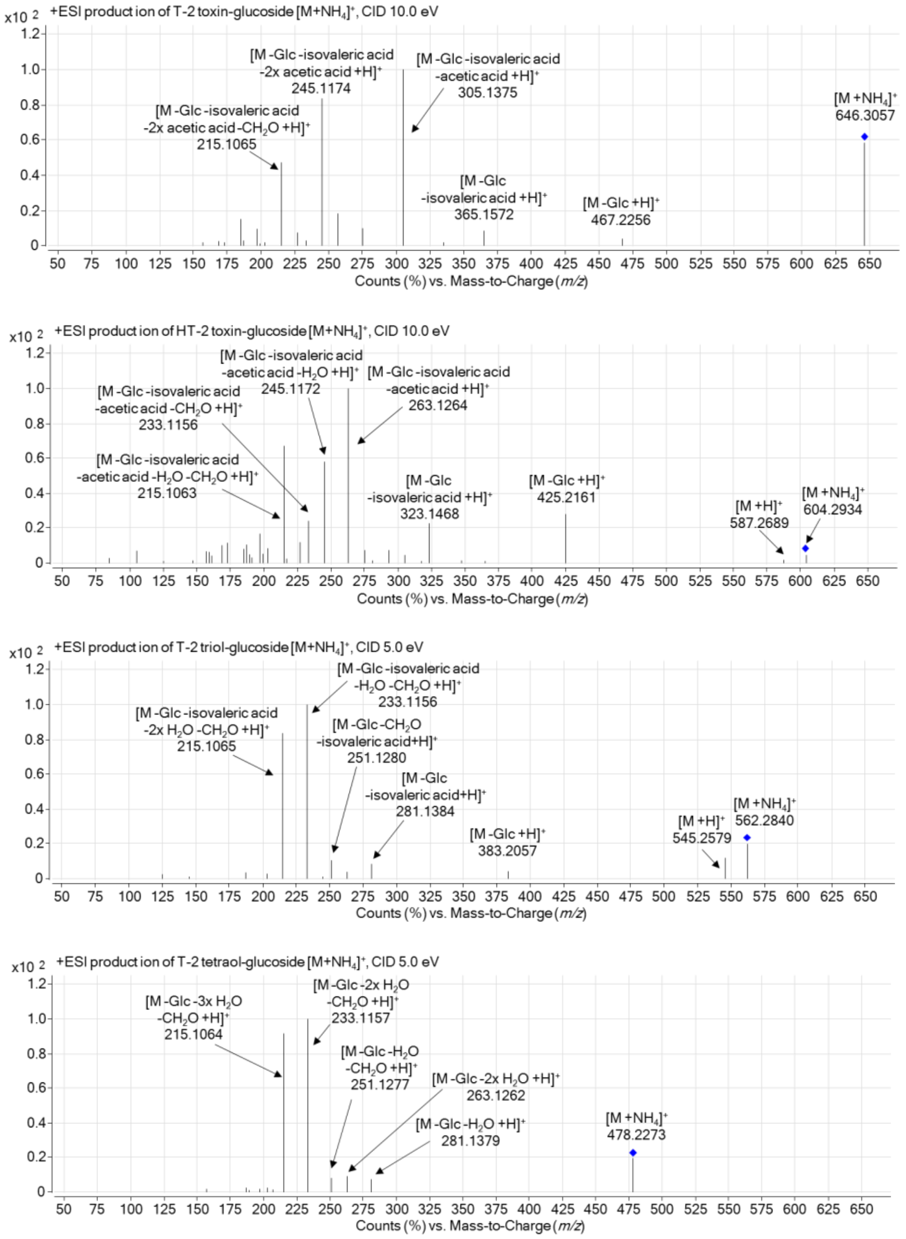

Figure S1. High resolution product ion spectra of the ammonium adduct of T-2 toxin-glucoside, HT-2 toxin-glucoside, T-2 triol-glucoside and T-2 tetraol-glucoside. Abbreviations: +ESI, positive electrospray ionization; CID, collision induced dissociation; Glc, glucose moiety.

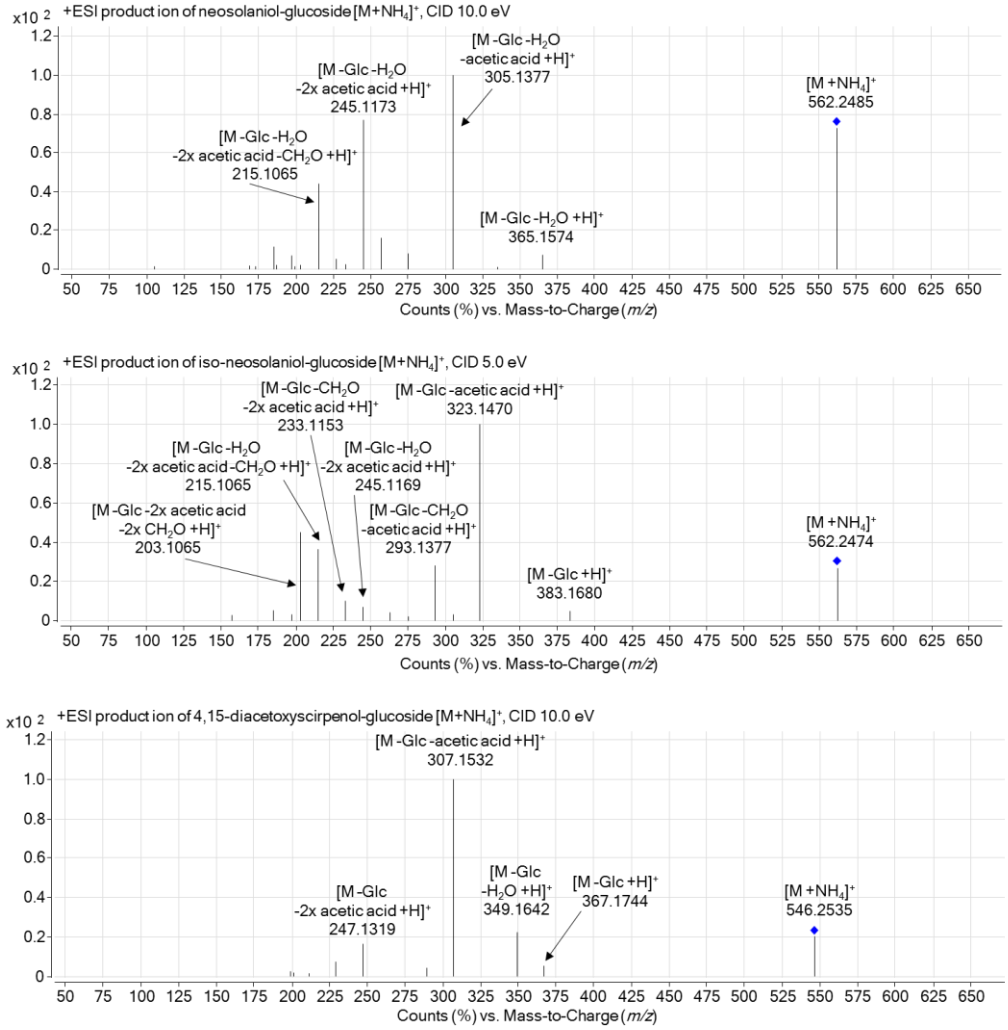

Figure S2. High resolution product ion spectra of the ammonium adduct of neosolaniol-glucoside, iso-neosolaniol-glucoside and 4,15-diacetoxyscirpenol-glucoside. Abbreviations: +ESI, positive electrospray ionization; CID, collision induced dissociation; Glc, glucose moiety

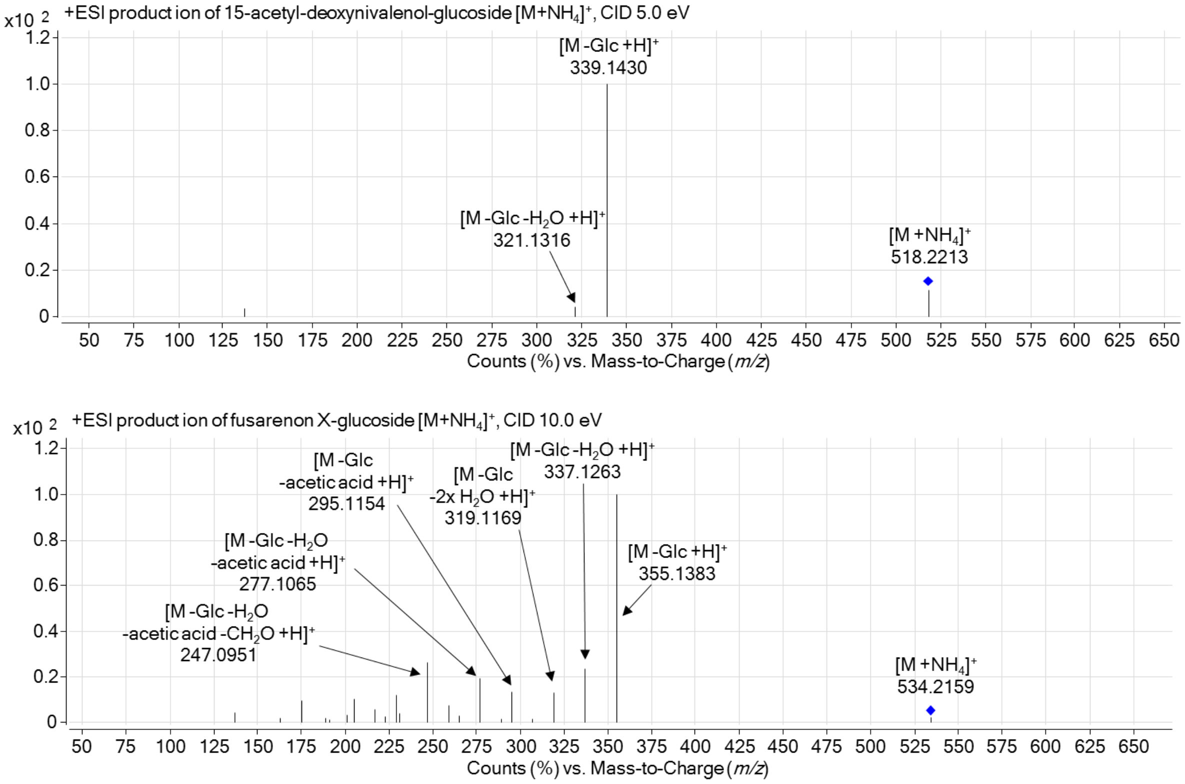

Figure S3. High resolution product ion spectra of the ammonium adduct of 15-acetyl-deoxynivalenol-glucoside and fusarenon X-glucoside. Abbreviations: +ESI, positive electrospray ionization; CID, collision induced dissociation; Glc, glucose moiety.

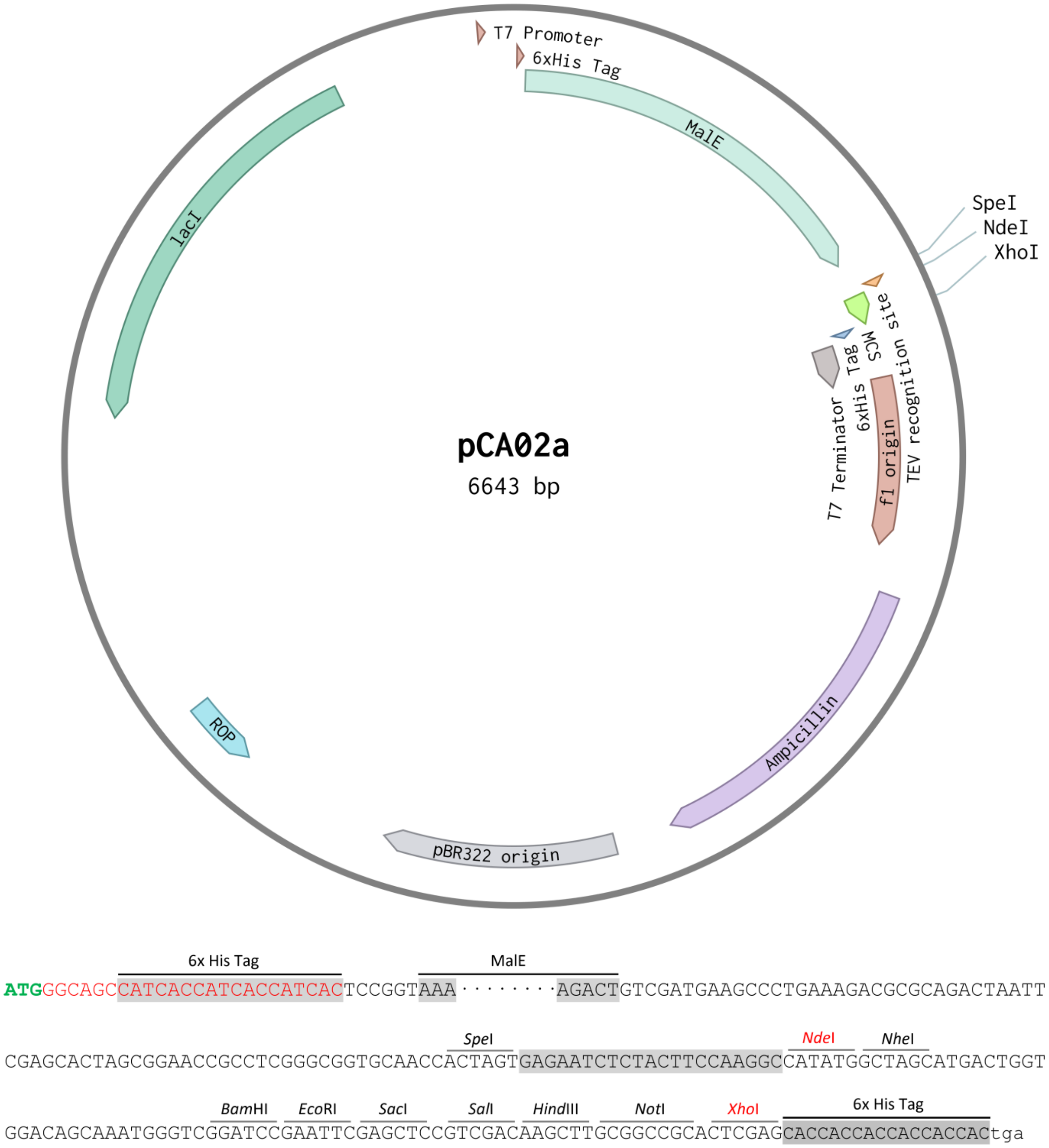

Figure S4. Topology of pCA02a/b. pCA02a is a derivative of pKLD116 [1] which in turn is a derivative of pET21a. pCA02a/pKLD116 are designed to express fusion proteins with *N*-terminal His$_6$-tag, maltose binding protein (MalE gene), TEV recognition site and the *C*-terminal target protein. pCA02 contains the multiple cloning site of the pET21 vector series. In pCA02b, the *N*-terminal His-tag (red sequence) was eliminated, the *C*-terminal His$_6$-tag is present in both pCA02a/b. The plasmid map was created with Benchling (https://benchling.com/).

References

1. Rocco, C.; Dennison, K.; Klenchin, V.A.; Rayment, I.; Escalante-Semerena, J. Construction and use of new cloning vectors for the rapid isolation of recombinant proteins from *Escherichia coli*. *Plasmid* **2008**, *59*, 231-237.
